# Found at Last

**Published:** 1874-01

**Authors:** 


### Editorial.

### FOUND AT LAST—

"*The Birthplace of Modern Dentistry*," for which, see editorial in Dec. No. of the *American Journal of Dental Science*, page 373.

We are glad this matter is settled so clearly and definitely. This diminishes by one the number of difficult, obscure and important subjects with which the profession has had, it would seem almost hopelessly, to struggle for these long years. The adjustment of this may open the way for the right disposition of other all-absorbing subjects. It may not be amiss to refer to one or two little mistakes, that appear in the historical notice referred to. It says, "In 1826, the 'Principles of Dental Surgery,' appeared in London, a work written by Leonard Kocker, a Baltimore physician."

The work referred to was written in London, by a man in practice there, and dedicated to David D. Davis, M. D., M. R. S. L., member of the Royal Colleges of Physicians of London and Edinburgh, etc., etc. Baltimore was not the birthplace of this work.

On same page, "This was followed in 1839, by the establishment in Baltimore of the American Journal of Dental Science." Now either the writer did not know the facts in the case, or, at any rate, he misstated them.

The organization for the publication of the Am. Journal was effected in New York; the publishing committee and one of the editors, Dr. E. Parmly, resided there. Dr. C. A. Harris resided in Baltimore; the Journal was printed in New York, and the prospectus says "published simultaneously in Philadelphia, Balimore, New York and Boston:" so that so far as the birthplace of the American Journal of Dental Science is concerned, it is similar to him who was "born at Nantucket, Cape Cod and all along the shore."

Whether the period alluded to, viz., 1839—40 was the precise period of the birth of the Dental profession may be judged by a few extracts from the prospectus of the first volume of the Am. Journal of Dental Science issued at *New York*, on the 1st day of June, 1839, by Drs. Eleazer Parmly, Elisha Baker and Solyman Brown, Publishing Committee, all of New York:

"1st, There is no *fraternity* in the *United States* of *equal magnitude* and *importance* which has not some publication devoted to its interest."

"There are many high-minded men of great knowledge and experience in our Art, who will gladly communicate the results of their observation for the benefit of the younger members of the *profession*."

"The publication of such a Journal will have the effect of giving dignity and inportance to the general subject of Practical Dentistry, and thus result in solid advantage to each and all of its professors, as well as to the community at large."

"This Journal will perpetuate those numerous treatises on Dental Science and Practice, which are now nearly out of print, as well European as American."

Now, we submit that it was highly improper for those gentlemen to talk in this style about a profession that was not yet born, and it is doubtful whether it entered their minds that the profession about which they spoke so freely was only an embryo. Isaac Greenwood practiced dentistry in Boston about the year 1775, John Greenwood, son of Isaac, practiced dentistry in New York about and subsequent to the year 1790. Many others soon after this entered upon the practice of dentistry, and there were in practice in 1839 and before the issue of the first No. of the "American Journal of Dental Science," such men as E. Parmly and seven other members of the Parmly family, Horace H. Hayden, Robt. Arthur, J. F. N. Flagg, J. W. Foster, John Allen, C. A. Harris and three hundred and forty others "good men and true" who were active and honorable practitioners of dentistry, and pledged their support to the Journa l There were hundreds of others occupying honorable positions in the profession throughout the country.

Now, that dentistry was born four thousand years ago or more, in Egypt, cotemporaneous with the builders of the Pyramids, we will not deny, for we have not much data pro or con upon that subject. But that it was *born again* at Baltimore about the year 1840, we do not believe. Now, we have no *special* preference as to the birthplace of dentistry ; indeed, "don't care a fig." Don't believe it was *born* at all, "*it just growed.*"

